# eHealth implementation *in Europe*: a scoping review on legal, ethical, financial, and technological aspects

**DOI:** 10.3389/fdgth.2024.1332707

**Published:** 2024-03-08

**Authors:** Britt E. Bente, Anne Van Dongen, Ruud Verdaasdonk, Lisette van Gemert-Pijnen

**Affiliations:** ^1^ Centre for eHealth and Wellbeing Research, Department of Psychology, Health and Technology, Faculty of Behavioural, Management and Social Sciences, University of Twente, Esnchede, Netherlands; ^2^ Section of Health, Technology and Implementation, Technical Medical Centre, University of Twente, Enschede, Netherlands

**Keywords:** implementation, eHealth, technology, healthcare, legal, ethical, business model, stakeholder engagement

## Abstract

**Background:**

The evolution of eHealth development has shifted from standalone tools to comprehensive digital health environments, fostering data exchange among diverse stakeholders and systems. Nevertheless, existing research and implementation frameworks have primarily emphasized technological and organizational aspects of eHealth implementation, overlooking the intricate legal, ethical, and financial considerations. It is essential to discover what legal, ethical, financial, and technological challenges should be considered to ensure successful and sustainable implementation of eHealth.

**Objective:**

This review aims to provide insights into barriers and facilitators of legal, ethical, financial, and technological aspects for successful implementation of complex eHealth technologies, which impacts multiple levels and multiple stakeholders.

**Methods:**

A scoping review was conducted by querying PubMed, Scopus, Web of Science, and ACM Digital Library (2018–2023) for studies describing the implementation process of eHealth technologies that facilitate data exchange. Studies solely reporting clinical outcomes or conducted outside Europe were excluded. Two independent reviewers selected the studies. A conceptual framework was constructed through axial and inductive coding, extracting data from literature on legal, ethical, financial, and technological aspects of eHealth implementation. This framework guided systematic extraction and interpretation.

**Results:**

The search resulted in 7.308 studies that were screened for eligibility, of which 35 (0.48%) were included. Legal barriers revolve around data confidentiality and security, necessitating clear regulatory guidelines. Ethical barriers span consent, responsibility, liability, and validation complexities, necessitating robust frameworks. Financial barriers stem from inadequate funding, requiring (commercial) partnerships and business models. Technological issues include interoperability, integration, and malfunctioning, necessitating strategies for enhancing data reliability, improving accessibility, and aligning eHealth technology with existing systems for smoother integration.

**Conclusions:**

This research highlights the multifaceted nature of eHealth implementation, encompassing legal, ethical, financial, and technological considerations. Collaborative stakeholder engagement is paramount for effective decision-making and aligns with the transition from standalone eHealth tools to integrated digital health environments. Identifying suitable stakeholders and recognizing their stakes and values enriches implementation strategies with expertise and guidance across all aspects. Future research should explore the timing of these considerations and practical solutions for regulatory compliance, funding, navigation of responsibility and liability, and business models for reimbursement strategies.

## 1 Introduction

Over the years, the development of eHealth technologies has revolutionized healthcare (1), providing substantial support to both patients and healthcare professionals (2). Initially serving as information and communication platforms, these technologies offered general disease descriptions and healthcare-related resources. However, with the advancement of knowledge and technical capabilities, these technologies have evolved into skill training platforms, empowering patients to actively monitor their health data, while healthcare professionals remotely monitor the patients' input. Furthermore, the scope of eHealth technologies has expanded to encompass treatment- and diagnoses-driven platforms, exemplified by the provision of additional exercises in mental healthcare and the utilization of patient-entered data to aid consultations and support healthcare professionals' decision-making for diagnoses (1).

The continuous expansion of knowledge and technical possibilities has resulted in increased automation and digitalization of data exchange within healthcare systems (3). Integration of eHealth technologies with existing health information and communication (ICT) systems, such as electronic health records (EHR), coupled with the use of artificial intelligence (AI), has yielded a multitude of benefits. These include enhanced interoperability (4, 5), the ability to reuse data (6, 7), streamlined workflows (8), improved decision-making support (9–11), and personalized care (1, 12). As our exploration of technological possibilities deepens and our focus on optimizing healthcare goals intensifies, it becomes evident that the scope of eHealth extends beyond standalone tools or platforms (13, 14). Rather, there is a paradigm shift towards the development of an all-encompassing environment that enables the integration of diverse eHealth tools and facilitates connections and networks among various healthcare stakeholders, including patients, healthcare professionals, pharmacies, and insurers. This environment necessitates extensive data exchange among multiple healthcare institutions and diverse health ICT systems (4). Consequently, these environments are no longer confined to static, tangible platforms, but encompass an overarching ecosystem that nurtures existing relationships, both physical and digital, and enables the exchange of data—a true digital health environment.

However, the development and implementation of digital health environments encounter notable challenges regarding privacy protection and medical-ethical considerations (1, 15, 16). The introduction of stricter legislations and regulations, including the Medical Device Regulations (MDR) (17), has amplified the scrutiny in these domains (15). Complying with these regulations entails meticulous attention to data security, informed consent, and safeguarding sensitive patient information. Similarly, the General Data Protection Regulation (GDPR) (18) and its national counterparts, impose stringent standards for the collection, storage, and processing of personal data, including health-related information. Upholding these regulations pose additional challenges for the implementation of digital health environments (3, 16, 19), as healthcare organizations must ensure legal and ethical requirements and maintain robust data protection measures. Consequently, these regulations can impede or even hinder the implementation progress of promising healthcare innovations.

Existing theoretical frameworks in the field of eHealth, such as the Non-Adoption, Abandonment, and Challenges to Scale-up, Spread, and Sustainability (NASSS) framework (20), have given limited attention to the exploration of legal and ethical dimensions in the implementation process of complex eHealth technologies, as Digital Health Environments (13, 21–23). These frameworks have primarily focused on technical and organizational factors (23), neglecting the intricate legal and ethical landscape that accompanies the integration of digital health technologies that impact multiple levels (such as individual, organizational, society) (14). Consequently, a significant knowledge gap exists in comprehending the legal and ethical implications of implementing such more advanced health technologies, particularly digital health environments. In response, our study aims to bridge the gap between the conventional medical models (such as NASSS) and the sociological perspectives on eHealth. By grounding sociological perspectives in structures that can embed their “ideals” into practice, we strive to provide a comprehensive understanding of the legal and ethical dimensions influencing the implementation of advanced health technologies. Practical experiences have underscored that a majority of challenges encountered in eHealth implementation arise from infrastructure-related barriers, encompassing legal, ethical, and financial dimensions (2, 23). For example, establishing interoperability among disparate healthcare systems and ensuring secure data exchange while adhering to legal requirements present substantial hurdles (24). Furthermore, addressing ethical considerations such as patient autonomy, consent, and privacy, further complicates the implementation process (25).

In addition, ensuring sufficient funding for the long-term maintainability and scalability of digital health environment is of utmost importance (26). Without a viable business model, these implementations may encounter challenges (27, 28), including limited resources for infrastructure maintenance, system upgrades, and data security measures. Inadequate funding can result in operational inefficiencies, suboptimal user experiences, and the inability to adapt and expand in response to evolving healthcare needs. Moreover, insufficient financial planning can impede the widespread adoption and utilization of digital health environments, constraining their potential to enhance healthcare delivery and outcomes. Therefore, a comprehensive understanding of the financial aspects and the development of appropriate business models are imperative for the successful implementation of complex digital health environments (3, 14, 26, 27).

The dynamic and evolving nature of legislation related to legal, ethical, financial, and technological aspects further complicates the implementation of digital health environments. To address these challenges and provide clarity on the considerations, this study aims to provide insights into barriers and facilitators of legal, ethical, financial, and technological aspects for successful implementation of multifaced eHealth technologies, which impact multiple levels and multiple stakeholders. Specifically, the study seeks to answer the following questions:

1. Which barriers and facilitators have been reported on the legal, ethical, financial, and technological aspects of eHealth technology implementation?
2. What lessons can be learned from the identified barriers and facilitators on legal, ethical, financial, and technological aspects of eHealth technology implementation?

Studying these aspects is evident in the inherent importance of safeguarding privacy, upholding ethical standards, and ensuring financial sustainability within digital health environments. Moreover, this study adopts a scoping review methodology to comprehensively explore the available evidence from a wide range of sources, allowing for a holistic understanding of the topic. This approach enables to provide practical recommendations for stakeholders and facilitate evidence-based decision-making and contributes to the advancement of successful implementation strategies for digital health environments. Our research is part of a larger consortium with the overarching objective of developing, evaluating, and implementing a trans-diagnostic and personalized eHealth platform (29). The focus of our work is specifically directed towards the examination of legal, ethical, financial, and technological aspects pertaining to the implementation process. Simultaneously, other consortium work packages address organizational and human factors. The insights gained from our study will be enriched by findings on these factors and will contribute as foundational elements essential for the formulation of a comprehensive roadmap guiding successful eHealth implementation.

## 2 Methods

This scoping review is reported considering the PRISMA-ScR (Preferred Reporting Items for Systematic Reviews and Meta-analyses extension for Scoping Review) checklist, without a prior registered review protocol (30). The review was designed by a multidisciplinary research team compromising eHealth experts in the field of development, implementation, and evaluation.

### 2.1 Eligibility criteria

Studies were included if (1) they described the development and/or implementation process of an eHealth technology, (2), they describe one, or more, of the following aspects of implementation: legal, ethical, financial, or technological aspects, and (3) the eHealth technology facilitates data exchange between users and/or systems. A more detailed description of the eHealth development and implementation process can be found in Table 1. Consciously, we chose to expand our focus to cover a range of eHealth technologies, as opposed to limited it to digital health environments, which are relatively novel and lack of documentation of implementation experiences. Valuable insights can be drawn from the well-established eHealth technologies like apps and platform, provided they align with our defined criteria. An eHealth technology that enables data exchange between users and/or systems is defined as any eHealth technology that allows users, such as patients or healthcare professionals, to input or retrieve health or treatment-related information. For instance, this included patients logging their health data or disease-specific information, or healthcare professionals providing additional information or treatments outside of clinical consultations. Additionally, it encompasses technologies capable of retrieving or inputting data from other (healthcare) systems, such as EHR. To account for differences in laws and regulations between Europe and other continents, studies published by a first author with a non-European affiliation, as well as studies conducted in non-European countries, were excluded. Furthermore, papers that solely presented user expectations, perceptions, and opinions before utilizing the technology were excluded. Evaluation studies that solely reported clinical outcomes regarding the effectiveness and impact of the eHealth technology without discussing the implementation process were excluded. However, studies employing a formative evaluation approach [defined as “activities throughout the entire development process that provide ongoing information on how to improve the development process, outcomes of activities and eHealth technology” (31)] were included, as these evaluations are intertwined throughout process of developing and implementing eHealth technologies. Studies conducted outside of a healthcare setting, as well as review articles and abstracts; were excluded. We opted to exclusively consider studies published after 2018 due to the enactment of the MDR (18), and given the significant transformations in the realm of big data and AI, consequential shifts in legal and ethical paradigms have emerged in the recent years.

**Table 1:** In- and exclusion criteria.

Inclusion criteria	Exclusion criteria
*Development* studies [entailing both the design phase (i.e. the functional creation of the health technology)] and *implementation* studies (i.e. activities to realize the introduction, adoption, dissemination and long-term use of a product in its intended context), including *formative evaluations* (i.e. activities throughout the entire development process that provide ongoing information on how to improve the development process, outcomes of activities and eHealth technology) (31)	Studies published outside Europe (i.e., studies of which the first author had a non-European affiliation, or the study was conducted in a non-European country)
Legal, ethical, financial, and technological aspects of implementation (one, or more of the aspects)	User expectations, perceptions, or opinions prior to usage of technology (i.e., concerns from users on guaranteeing privacy/security before they have actually experienced and used the technology)
eHealth technologies that facilitate health or treatment related data exchange between users and/or systems	Evaluation studies (i.e., showing only clinical outcomes, such as effectiveness studies, without reporting on the implementation)
	Wrong setting (i.e., not in a healthcare setting)
	Published ≥2018
	Unavailable in English
	Full-text not available
	(Poster) abstracts (incl. brief reports)
	Non-primary data (e.g., systematic reviews)

### 2.2 Information sources, search and selection of evidence

A comprehensive and systematic literature search encompassing PubMed, Scopus, Web of Science, and ACM Digital Library, without language restrictions, was performed. Given the rapidly evolving nature of eHealth development and implementation, and legal and ethical regulation, only studies published in or after 2018 were considered. On September 13, 2022, reviewer BB conducted the initial search in all databases, and a subsequent update search was executed on September 12, 2023. A structured query, designed in collaboration with eHealth experts and an information specialist, was applied to all four databases. This query was constructed comprising the following terms: ([(“*health technology*” OR “*e-Health*” OR “*electronic health*” OR “*digital health*” OR “*digital platform*” OR “*mobile health*” OR “*telehealth*” OR “*telemedicine*” OR “*telemonitoring*” OR “*mobile application*”)] AND (Implement* OR adopt* OR “*daily practice*”)) AND (*legal* OR *law* OR regulat* OR *privacy* OR ethic* OR validat* OR certificat* OR financ* OR “*business model*”). In the initial screening round (encompassing studies published between 2018 and September 2022), the Covidence web-based software platform was used to remove duplicates and facilitate a meticulous evidence selection process. This process involved title and abstract screening (BB and AD), as well as full-text screening (BB, AD, RV, LGP), with conflicts resolved through consensus. For the search update, which considered studies published from September 12, 2022, to September 13, 2023, the AI tool “ASReview” (V.0.17.1) (32) was employed to screen titles and abstracts, a method successfully employed in previous studies (32–35). ASReview utilizes an active researcher-in-the-loop machine learning algorithm, employing text mining techniques and multiple classifier models to rank studies based on their eligibility for inclusion. The algorithm was trained using the entire assessed dataset from the initial round, with all studies labeled with “ASReview_relevant” and “ASReview_irrelevant”, while the studies identified during the search update were labeled as “ASReview_not_seen”. This labeled dataset was considered by ASReview to generate a ranking of the non-assessed studies. Subsequently, the top-ranked studies were presented to reviewer BB, who determined their eligibility for inclusion. This iterative process, wherein the AI system ranked the studies and the reviewer made eligibility decisions, continued until a predefined data-driven stopping criteria of 200 consecutive irrelevant studies was met. Studies labeled as relevant during the title and abstract screening underwent independent full-text screening by the research team, mirroring the approach used in the initial round.

### 2.3 Data extraction and charting

The general study characteristics extracted were first author, country, year, journal, study aims, study design and methods, and the healthcare setting. Given the purpose of this study, which aims to provide insights into barriers and facilitators, an open data-charting form was created. This form structured to categorize the identified barriers and facilitators into the specific domains, namely legal, ethical, financial, and technological. The main researcher (BB) read all full texts and systematically extracted key points that mentioned barriers and facilitators regarding the legal, ethical, financial, and technological aspects of the implementation of eHealth. All relevant fragments were extracted and summarized in tables. The identified barriers and facilitators were merged into topics via an iterative axial and selective coding process by BB. The data extraction form was discussed within the research team, and iteratively refined throughout the extracting process.

## 3 Results

### 3.1 Study selection

**Figure 1:**
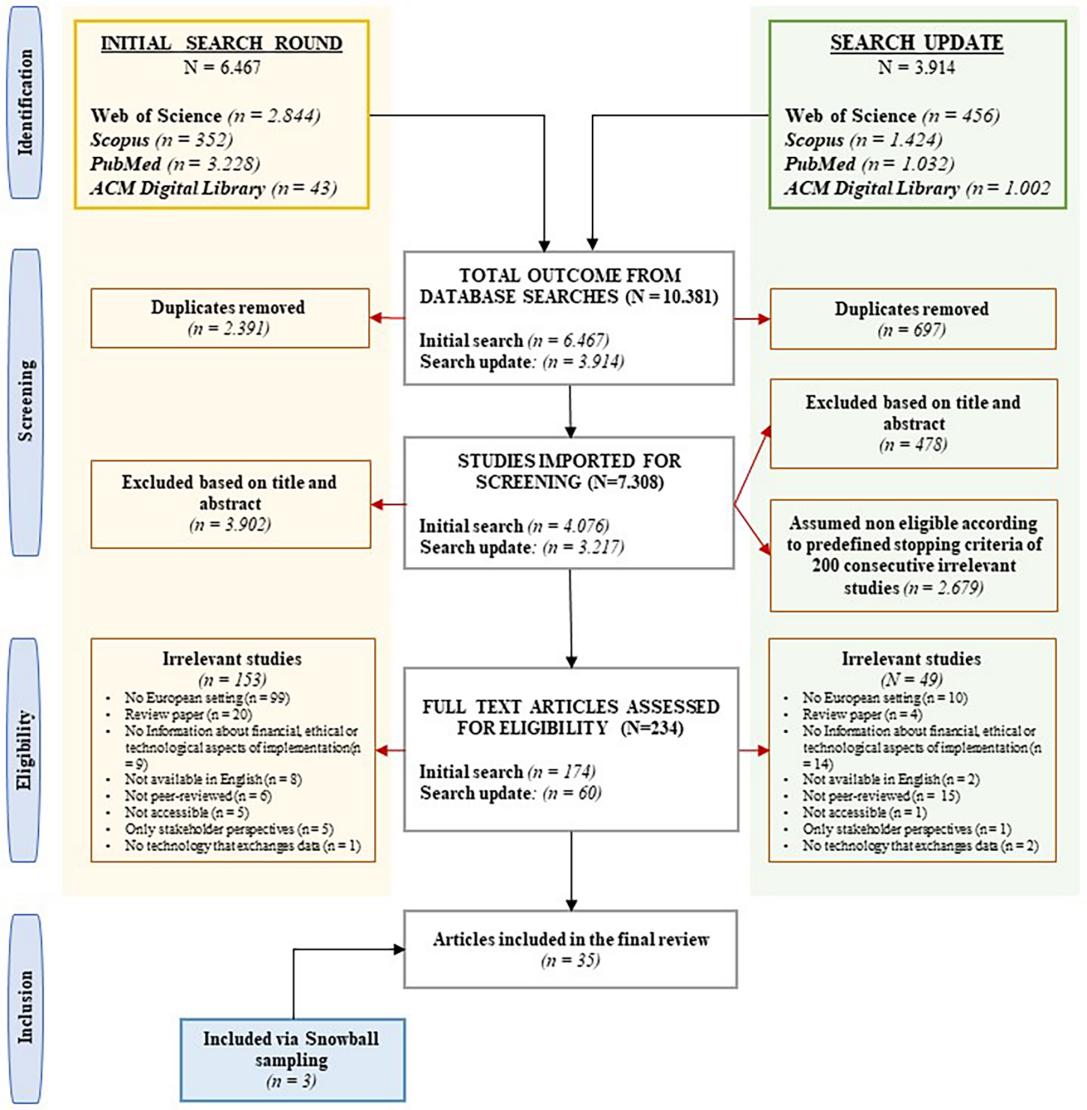
PRISMA (Preferred Reporting Items for Systematic Reviews and Meta-Analyses) flowchart of included and excluded studies, including reasons for exclusion.

In the initial literature search, 6,467 potentially relevant abstracts were identified. Following the removal of 2,391 duplicates (36.97%), 4,076 unique titles and abstracts (63.03%) were subjected to assessment. This led to the eligibility assessment of 174 (2.69%) full texts. The search update yielded 3,914 potentially relevant abstracts. After eliminating 697 duplicates (17,81%), 3,217 unique titles and abstracts were imported for screening. Among the 478 articles (14.86%) assessed during the title and abstract screening, the ASReview process halted upon reaching the predefined data-driven criteria of 200 consecutive irrelevant studies. This led to the eligibility assessment of an additional 60 full texts (1.87%). Ultimately, 35 studies were included, including an additional three identified through snowball sampling. The primary reasons for exclusion were non-European settings, review studies, insufficient information about legal, ethical, financial, or technological aspects of implementation, and non-peer-reviewed articles. See Figure 1 for a flowchart of the study selection.

### 3.2 Study characteristics

In total, this review included 35 papers published between 2018 and 2023, with a predominant representation from Web of Science and PubMed databases. The studies primarily originate from the United Kingdom, Germany, The Netherlands, and Italy, with contributions from various multidisciplinary journals spanning digital health, health policy, health-medical informatics, and medical ethics. The included studies encompass an extensive range of eHealth technologies, encompassing diverse domains. These technologies include AI and machine learning systems, EHR, digital health technologies in various contexts (e.g., mental health, palliative care, health insurers’ apps, apps on prescription), and patient remote monitoring systems. Additionally, some studies do not focus on a particular eHealth technology but focus on broader regulatory frameworks for health technologies. The included studies explore these technologies or regulations through mainly qualitative methods, including stakeholder workshops, surveys, interviews, and case studies. Topics covered span a broad range of areas within healthcare implementation and eHealth adoption. Most studies focus on integrating technologies, such as AI and digital health platforms, into healthcare systems. The studies address ethical concerns, regulatory frameworks, privacy and security considerations, and the wider implications for healthcare stakeholders and systems. Collectively, these studies aim to offer an extensive perspective of the challenges, opportunities, and considerations in the dynamic landscape of eHealth implementation and the digitization of health services. In Table 2, an overview can be found of the included studies and their characteristics.

**Table 2:** Characteristics of the included studies.

#	Author and country	Year	Journal	Study design and methods	Type of eHealth technology	Topics related to implementation
1	Bahls et al, Germany (36)	2020	Journal of Translational Medicine	**Qualitative**: - Interdisciplinary workshops with family doctors, experienced researchers, medical data managers, software architects and developers, data security experts and Technology, Methods, and Infrastructure for Networked Medical Research staff - Practices of family doctors are visited and discussions with staff were held	A generic architecture with a technical framework of tools, interfaces, and workflows for practicable and secure processing of patient data family doctors	Information about the design, implementation, and pilot testing of this generic research architecture and workflows that unlock primary care data for secondary usage
2	Botrugno et al, Italy (37)	2018	Health Policy and Technology (Elsevier)	*Not specified*	Telemedicine in general	Information about exploring available provisions for an EU regulatory framework for telemedicine, and to assess their suitability to regulate remote care services
3	Briganti et al, Belgium (38)	2020	Frontiers in Medicine	*Not specified*	Artificial Intelligence in healthcare	Information about the benefits, future opportunities, and risks of established AI applications in clinical practice on physicians, healthcare institutions, medical education, and bioethics
4	Cobianchi et al., Italy (39)	2022	Journal of the American College of Surgeons	**Qualitative**: – Stakeholder workshop with experts in the fields of academic surgery, radiology, surgical ethics, AI and Machine-learning, computer sciences, innovation, strategy, business models, and healthcare policies	Artificial intelligence (AI) applications aiming to support surgical decision-making processes	Information about mostly ethical (but also legal, technological, and business model related) factors that need to be considered when implementing AI applications to support surgical decision-making. These include factors related to human agency and oversight, technical robustness, privacy, and safety of data, diversity, and non-discrimination, societal and environmental well-being, and accountability.
5	Cresswell et al, United Kingdom (40)	2019	BMJ Health & Care Informatics	**Qualitative**: - Complementing recent work conducted by the American Medical Informatics Association (AMIA) exploring potential policy frameworks and associated strategies for Patient Generated Health Data	Integration of patient generated data into electronic health records	Information about applying emerging policy frameworks to the United Kingdom and outline five key priority areas that are intended to guide policymakers’ decision-making for patient generated data integration into electronic health records
6	Diaz-Skeete et al, Ireland (41)	2020	Health Informatics Journal	**Qualitative**: - Interactive workshop with clinicians, academic researchers, technologists, patient advocates, policy makers, and representatives from the health service	eHealth technologies (in particular remote monitoring systems) in community and home cardiac care	Information about the barriers and facilitators to the adoption of eHealth technology in community and home cardiac care
7	Gaebel et al, Germany (42)	2020	European Archives of Psychiatry and Clinical Neuroscience	**Mixed-method**: - Narrative review (status analysis of 6 countries) - Qualitative stakeholder interviews (52 interviews with experts in science, politics, small-to-medium enterprises, care providers, and patients	e-Mental Health Innovations	Information about the uptake of E-mental health, including barriers for implementation (for six different countries). Policy recommendations were provided to guide political and regulatory processes regarding the implementation of e-mental health.
8	Garani-Papadatos et al., Greece (43)	2022	Frontiers in Digital Health	**Qualitative**: - Ethical exploration of specific eHealth technology (MyPal4Kids)	A digital health platform in a palliative care context (pediatric cancer patients)	Information about ethical challenges, the paper provides information about what ethical considerations occurred in the development and implementation of this digital health platform, and what type of decisions they made (e.g., in terms of technological features) to tackle the barriers they explored.
9	Gilbert et al., Germany (44)	2023	Journal of Medical Internet Research	**Qualitative**: - Multistakeholder interactive workshops with healthcare professionals, regulators, healthcare providers, healthcare system digitization bodies, regulatory science and law academics, regulatory specialists, and consultants	Digital health technologies, particularly AI-based tools and machine learning medical tools	Information about the divergent approaches of the European Union and the United States in the implementation of new regulatory approaches in digital health. The paper poses specific challenges to the development and implementation of functional regulation (such as the MDR).
10	Jacquemard et al, Ireland (45)	2021	BMC Medical Ethics	**Qualitative**: - Re-examination of literature to determine ethical challenges and opportunities - Workshops with multidisciplinary stakeholders (ethicist, senior hospital-based physician, eHealth expert) to discuss and reach consensus on interpretation of the literature	Electronic health records	Information about ethical concerns that occur in the design, development, implementation, and use of an electronic health records
11	Jusob et al., United Kingdom (46)	2022	Journal of Public Health	**Qualitative**: - Modified version of the engineering design process	A novel privacy framework to address privacy threats/concerns in the context of mHealth and the management of chronic diseases	Information about possible privacy threats and concerns in the development and implementation of mHealth. The paper proposes a new privacy framework for mHealth, in which they describe requirements for the framework and the choices they made based on these requirements.
12	Karacic Zanetti and Nunes, Croatia (47)	2023	Computers	**Qualitative**: - Investigation of health wallets’ specific features and capabilities	Health wallet *(an integrated digital platform that allows individuals to manage and control their own health information)*	Information about the implementation of health wallets. In particular a risk-based approach to identify and prioritize data security risks, system interoperability, cross-border healthcare challenges, data accuracy, and ethical challenges. The paper provides a practical framework for healthcare organizations to allocate resources while ensuring that patient data remain secure and private.
13	Kühler et al, France (48)	2022	Clinical Therapeutics	**Qualitative**: - Working group with experts from pharmaceutical, medical device, and tech companies	Not a specific eHealth technology, but the paper focusses on European Union regulatory frameworks for connected combined products, such as medical devices	Information about identified challenges in developing and releasing connected combined products and the paper highlights and discusses gaps in the European Union Regulations.
14	Leimanis et al, Latvia (49)	2021	European Journal of Sustainable Development	**Qualitative**: - Considering of international legal regulations	Artificial Intelligence in healthcare	Information about ethical, regulatory, and social issues raised by applying artificial intelligence in healthcare, from sustainable development perspective
15	Li et al., United Kingdom (50)	2023	BMC Medical Informatics and Decision Making	**Qualitative**: - Interview study	Electronic Health Records (EHR)	Information about (the lack of) EHR interoperability, including facilitators and barriers to improve EHR interoperability.
16	Martani et al, Switzerland (51)	2019	Swiss Medical Weekly	**Qualitative**: - Field research into all insurers’ data-sharing apps on the Swiss Market	Insurers’ apps that permit customers to share their data in exchange for monetary rewards	Information about the features and functioning of the apps, and ethically relevant aspects related to the usage of these apps.
17	Parimbelli et al, Italy (52)	2018	International Journal of Medical Informatics (Elsevier)	**Qualitative**: - Workshops with system developers, researchers, physicians, nurses, legal experts, healthcare economists, and administrators	Telemedicine systems, with the focus on real-world, non-mediate interaction with the final user	Information about the risks and legal implications connected to the development and use of modern telemedicine systems.
18	Prodan et al, Germany (53)	2022	Frontiers in Digital Health	**Qualitative**: - Interviews with public bodies and industry - Multi-stakeholder workshop	Digital therapeutics (“apps on prescription”)	Information about how the approval and adoption of digital therapeutics within health systems have been approached in five selected European countries and regions, including success factors that scale up the adoption.
19	Rakers et al., The Netherlands (54)	2023	Elsevier Health Policy and Technology	**Qualitative**: - Interview study	Remote Patient Monitoring (RPM)	Information about the barriers and facilitators of structural reimbursement of RPM in hospital care in the Netherlands. In addition, the paper proposes actionable recommendations.
20	Rauwerdink et al, the Netherlands (55)	2021	Journal of Medical Internet Research	**Mixed method**: - Questionnaire - Semi-structured interviews	eHealth technologies with various subjects and themes, from the Citrien Fund program eHealth (2016–2019)	Information about the barriers and facilitators of the development of the 29 included eHealth projects
21	Redrup Hill et al., United Kingdom (56)	2023	Frontiers of Digital Health	**Qualitative**: - Multistakeholder interactive workshops	A semi-automated deep-learning system (AI) with as example the clinical pathway for the early detection of Barett's Oesophagus and oesophageal adenocarcinoma.	Information about ethical and legal considerations that influence human involvement in the implementation of AI in a clinical pathway. Opinions of stakeholders are shared about the risks and potential harms of AI, the impact of AI on human experts, equity and bias, transparency and oversight, patient information and choice, and accountability, moral responsibility, and liability for errors.
22	Reindl et al, Germany (57)	2021	IEEE Xplore	*Not specified*	Robot-assisted haptic telepresence tools	Information about the legal framework of telemedicine in EU, and practical physical safety, data security, and system usability implications learned from implementing a telemedicine station prototype.
23	Schlieter et al, Germany (58)	2019	Journal of Medical Internet Research	**Qualitative**: - 2-Round group workshop	Digital health innovations (in general)	Information about enablers and barriers for scaling up digital health innovations, in the context of achieving large-scale implementations that will benefit the population as a whole
24	Scobie et al, United Kingdom (59)	2020	Learning Health Systems	**Qualitative**: - Seminar, bringing together National Health Service leaders, academics, practitioners, and policymakers	Learning health systems	Information about requirements for the development of learning health systems, including national policy implications and actions.
25	Sheikh et al, United Kingdom (60)	2021	The Lancet Digital Health	*Not specified*	Learning health systems	Information about achieving the optimal balance between top-down and bottom-up implementation, improving usability and interoperability, developing capacity for handling, processing, and analyzing data, and addressing legal and ethical challenges.
26	Shull et al, Spain (61)	2019	JMIR Medical Informatics	*Not specified*	Digital health systems (and their interoperability with electronic health records)	Information about the evolution and obstacles of electronic health records, potential barriers for interoperability with digital health systems, and best practices from examples they provide.
27	Silven et al, the Netherlands (62)	2022	BMC Health Services Research	**Qualitative**: - Series of multidisciplinary focus groups with stakeholders who have relevant digital health expertise	Digital health in clinical practice	Information about challenges of responsibility and liability when prescribing digital health in clinical practice
28	Tozzi et al, Italy (63)	2021	BioLaw Journal	*Not specified*	Artificial Intelligence in healthcare	Information about potential ethical challenges and risks of implementing artificial intelligence tools in clinical practice, GDPR, and informed consent.
29	Van den Wijngaart et al, the Netherlands (64)	2018	Journal of Medical Internet Research	**Qualitative**: - Qualitative survey study	A web-based portal to monitor asthmatic children as a substitution for routine outpatient care	Information about barriers and facilitators for the implementation and use of this web-based portal
30	Van Rooden et al, the Netherlands (65)	2021	Clinical Microbiology and Infection	**Qualitative**: - 2 workshops of stakeholder discussions - A taskforce was installed that further elaborated governance aspects by reviewing documents and websites, consulting experts, and organizing teleconferences	Automated surveillance for healthcare-associated infections	Information about the governance aspects (both legal and ethical) of large-scale implementation of automated surveillance of infections
31	Van Velthoven et al, United Kingdom (66)	2019	Journal of Medical Internet Research	**Qualitative**: - Stakeholder workshop with patients, carers, local hospitals, pharmacy retailers, health insurers, health services researchers, engineers, and technology and pharmaceutical companies in Switzerland	Digital health innovations (in general)	Information about facilitators and barriers of sustainable adoption of digital health innovations, needs and expectations of stakeholders, and the safety, quality, and usability of the innovations.
32	Verweij et al., The Netherlands (67)	2022	BMC Health Services Research	**Qualitative**: - Case study of a digital care platform with in-depth interviews	A digital health platform for patients with chronic myeloid leukemia	Information about barriers and facilitators for the implementation of the digital health platform. In addition, a comprehensive implementation guide was developed for launching future digital care platforms in daily clinical platforms.
33	Wong et al, Switzerland (68)	2022	The Lancet Regional Health—Europe	*Not specified*	Digital health technologies (in context of effective surveillance systems in public health)	Information about the opportunities, challenges, and implications of the increasing digitalization of public health in Europe.
34	Zarif et al, United Kingdom (69)	2022	Springer/ Health and Technology	*Not specified*	Digital health innovations (in general)	Information about the ethical challenges that adoption of digital healthcare technology presents, contextualized at multiple levels, with suggested potential solutions.
35	Zemplényi et al, Hungary (70)	2023	Frontiers in Public Health	**Mixed method**: - Survey to rank predetermined barriers from literature - Stakeholder workshops to develop and review recommendations based on the barriers	Artificial Intelligence tools	Information about human factor related barriers, data related barriers, methodological barriers, regulatory and policy related barriers, and technological barriers of AI. In addition, this paper proposes recommendations on how to overcome the most important ones (based on a stakeholder ranking of the barriers).

### 3.3 Barriers and facilitators of implementation

**Figure 2:**
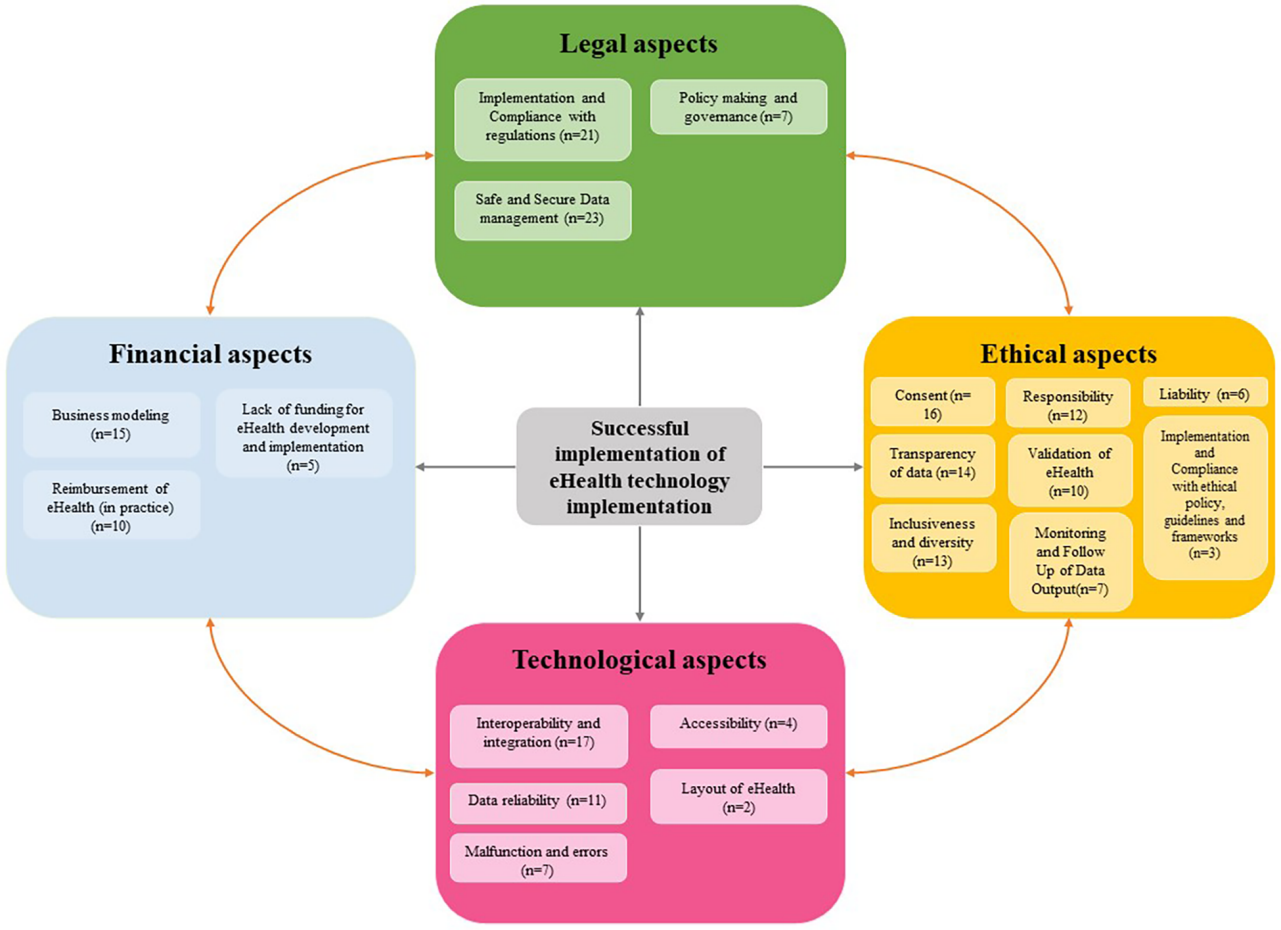
Overview of all domains and identified topics of barriers and facilitators.

In relation to each domain—legal, ethical, financial, and technological aspects of implementation—barriers and facilitators have been identified, which are elaborated upon below. Each paragraph provides detailed explanations regarding the specific barriers and facilitators within its domain, which are specified among subheadings per topic. Absence of entries for barriers or facilitators in the tables does not imply their nonexistence in that domain, but rather signifies their omission from the included literature. Figure 2 provides an overview of all domains and the identified topics of barriers and facilitators.

#### 3.3.1 Legal aspects

The included studies showed multiple legal barriers for implementation of complex eHealth technologies, with topics related to a safe and secure data management, guaranteeing data confidentiality, data storage, implementation and follow-up of regulations, and policy making and governance. See Table 3 for an overview of all identified barriers and facilitators of legal aspects.

**Table 3:** Overview of the identified **legal** barriers and facilitators.

Topic		
	Barriers	Facilitators
Safe and Secure Data Management (*n* = 23 studies)	Challenges in ensuring and maintaining data confidentiality (42, 45–47, 59, 63)	Establish robust security measures against data breaches (36, 37, 39, 45–48, 59, 60, 62, 63, 65)
	Threat of cyber-attacks (46, 47, 60, 63)	Employ advanced technologies for secure data storage and transmission (46, 60, 61, 63, 70)
	Challenges in ensuring safe storage and transmission of patient data (46, 57, 60)	Perform impact and risks assessments (40, 45, 47, 48, 55, 65)
	Difficulty in balancing competing interests in medical device regulation (44)	Ensuring privacy-preserving data sharing (36, 46, 70)
	Difficulty in balancing patient safety with advancements in healthcare (30)	Ensure health technology complies with data security and privacy regulations (47, 67)
	Exceptionalism imposing unrealistic AI standards, impeding adoption (56)	Empower patients as data owners (38, 47)
		Ensure a balance between data security and data liquidity promotion (40)
Implementation and Compliance with regulations (*n* = 21 studies)	Complex interpretation of legislative regulations hinders compliance (41, 42, 48, 57, 59, 67)	Provide a clear and understandable regulatory framework (42, 48, 49, 51, 57, 58, 60, 61, 65, 66)
	Stringent regulations impose restrictions in innovations (42, 44, 58)	Regularly assess and adapt governance structure and procedures to evolving developments (65)
	Current regulatory frameworks lack adaptability for various device types and operating techniques (44, 52, 56)	
	Data governance arrangements differ depending on the purpose of data (45, 59)	
	Although regulations for eHealth are fragmented across the EU, disparities exist between countries (47)	
	Diverse technology classifications require distinct regulatory strategies (48)	
	Ensuring compliance with the MDR's requirement to maintain device performance when used in combination with other technologies poses difficulties (48)	
	A regulatory framework for product connectivity remains unaddressed by the MDR (48)	
	Legislation lacks clarity on defining, developing, assessing, and managing risks in device component combinations (48)	
	Managing a substantial volume of sensitive information leads to regulatory compliance challenges (56)	
	Data protection regulations limit access to patient-level databases (56)	
	Risk and impact assessments cause planning delays (55)	
Policy making and governance (*n* = 7 studies)	Insufficient political commitment hinders effective policymaking and governance (70)	Develop data governance and ethical frameworks that promote data sharing (40, 45)
	Difficulty in balancing stringent regulations with inadequate oversight poses challenges in critical health sectors (44)	Promote awareness and political commitment (70)
		Incorporate provides perspectives alongside regulatory considerations in frameworks (53)
		Consider GDPR for crafting targeted legislation on data protection and interoperability (68)
		Establish a method for market entry approval (58)
		Include standardized catalog, indicator types, and accepted methods in evaluation procedures (53)
		Conduct a “risk assessment” of the regulations (44)
		Conduct a “conformity assessment” of the regulation by an independent entity (44)
		Implement post market surveillance to evaluate regulation performance (44)
		Perform Root Cause Analyses of problems that occur during the implementation of regulations (44)

##### 3.3.1.1 Safe and secure data management

The primary challenge in safe and secure data management is maintaining data confidentiality (42, 45, 47, 59, 63, 71). For example, even after anonymization, the persistent risk of re-identification remains, particularly through cross-matching and amalgamation of datasets (59, 63). The challenge of ensuring data confidentiality pertains not only to safeguarding patients' personal information, but also extends to the sensitive data of individuals in close proximity to the patient, necessitating personalized considerations (42). Cybersecurity threats, including privacy breaches and input manipulation, pose significant barriers (46, 47, 60, 63). To ensure the safe and secure management of data, several facilitating factors must be considered. Security protocols (36, 37, 39, 45–48, 59–63, 65), software updates (60), audit trials (45, 48), access control policies (45), the use of passwords or biometric authentication (62) are crucial. Additionally, implementing systematic procedures for data management and breach response strategies (60, 65), and incorporating targeted privacy safeguards for the transmission of sensitive information, both internally and externally (36, 60), are essential facilitators. Compliance with data security regulations, such as GDPR and MDR (47, 67) is imperative, and implementation can be facilitated by conducting impact and risk assessments, including Privacy Impact Assessments (55) and Data Protection Impact Assessments (65). Advanced technologies, such as the application of blockchain (61, 63), virtual local networks (60, 70), and secure cloud-based computing solutions (60) can enhance efficient and secure data storage and transmission. Incorporating a trusted third party for data management and pseudonym assignment is a potential facilitating measure (36).

##### 3.3.1.2 Implementation and compliance with regulations

From a research and development perspective, implementing eHealth technology faces a significant barrier in navigating complex and non-standardized legislative regulations (41, 42, 48, 57, 59, 67), causing uncertainties in interpreting norms such as MDR and GDPR within the context of eHealth technology (57, 67). Present regulations are highly restrictive (42, 44, 58), particularly the stringent MDR requirements for manufacturers, which can make compliance nearly impossible and stifle innovation within the EU (35, 44). Moreover, although the regulations for eHealth are fragmented across the EU, disparities exist between countries (47). Conversely, clear and understandable regulatory frameworks addressing key concerns (42, 48, 49, 51, 57, 58, 60, 61, 65, 66) can facilitate implementation. For example, the establishment of clear national legislation for development, market entry (58), and the security, privacy, and resilience against “hacking” of digital health innovations (66) proves essential. Ongoing evaluation and adaptation of governance structures in response to dynamic developments significantly facilitate implementation (65).

##### 3.3.1.3 Policy making and governance

From a policymaking and governance perspective, a challenge stems from a lack of strong political commitment. This deficiency is exemplified by the lack of a dedicated health digitization strategy and the failure to establish relevant databases (70). Striking a balance between stringent regulations and adequate oversight in healthcare is crucial for ensuring essential medical technologies while maintaining rigorous quality control (44). Facilitating implementation involves formulating data governance and ethical frameworks enabling data sharing (40, 45), establishing technology market entry approval methods (58), and expanding existing data protection frameworks to cover various patient-facing technologies and their associated health data (40). Regulations can support establishing a trusted environment for capturing and sharing personal health data (45). Evaluation procedures should incorporate a standardized, publicly available catalog of required evidence, indicator types, and approved methods (53). GDPR considerations are pivotal for crafting targeted legislation on data protection and interoperability (68). Implementation can be further facilitated through risk and conformity assessments, as well as root cause analysis on the regulation themselves, to ensure their practical applicability (27). Moreover, raising awareness and political commitment are essential (72), as is the incorporation of provider perspectives within regulatory considerations (53). Pertinent barriers and facilitators from the provider perspective were addressed in the preceding paragraph.

#### 3.3.2 Ethical aspects

The included studies showed multiple ethical barriers for implementation with topics related to consent, validation of eHealth, responsibility, liability, inclusiveness and diversity, monitoring and follow up of data output, ethical policy, guidelines and frameworks, and autonomy. See Table 4 for an overview of all identified barriers and facilitators of ethical aspects.

**Table 4:** Overview of the identified **ethical** barriers and facilitators.

Topic		
	Barriers	Facilitators
Consent (*n* = 16 studies)	Ethical concerns appear when patients are compelled to use a specific eHealth technology due to a lack of alternatives (47, 56)	Ensure transparent information disclosure to users (42, 43, 45, 49, 56, 58, 63, 65)
	Deleting data from users who have withdrawn consent may be impossible, especially when it is anonymized (43, 63)	Obtain explicit consent for data sharing (36, 45, 47, 49, 56, 63)
	Patient data may be shared with other providers or third parties without the patient's explicit consent (47)	Uphold users’ right to withdraw consent (43, 47)
	Ensuring the acceptance and consent of both patients and medical professionals is challenging (70)	Streamline the process of obtaining and managing consent (36, 53)
	Patients may lack the ability to provide consent in emergency situations (45)	Empower patients to control access to their data (47)
	Privacy concerns can arise when parents have access to their children's records (45)	Supply age-appropriate information to inform children, even if they lack legal capacity for consent (43)
	Concerns arise about obtaining patient consent for various treatment options (59)	Present information to patients in an easily understandable format to aid in data interpretation (45)
	The process of obtaining consent can be resource intensive (40)	
	Users often overlook the fine print or simply click on “agree” without reading it carefully (51)	
Transparency of data (*n* = 14 studies)	Opacity in the functioning of AI algorithms (39, 45, 56, 63)	Ensure transparency in data quality assessment (63, 65)
	Lack of awareness among patients about the storage and sharing of their (sensitive) data (51, 66)	Ensure transparency in the decision-making process based of AI data (39, 56)
	Insufficient methodological transparency in deep learning models (70)	Promote the development of “open source” health technologies (58, 65)
	The increasing complexity of algorithms leads to decreased decision support precision in earlier (older) models (39)	Engage all relevant stakeholders in decision-making, potential adoption, and discussions regarding data usage boundaries (53, 60)
		Enhance transparency in data infrastructure and data flow (67)
		Identify key stakeholders in the decision-making process for system and data-related matters (45)
Inclusiveness and diversity (*n* = 13 studies)	AI may contain biases that can unintentionally exclude or harm individuals (39, 56, 70)	Develop technologies that do not discriminate (43, 47, 49, 65)
	Inequity in access and use of healthcare technology (60, 69)	Create user-friendly software to enhance ease of use (47, 58, 60)
	eHealth technologies could potentially be used as an excuse to reduce the provision of high-quality care by trained health professionals (43)	Ensure that individuals facing particular needs or risks are encompassed by the social security system's protection (51)
	Favoring users who willingly share health-related data over those who do not share (51)	
	Ongoing monitoring and privacy violations can lead to increased stigma around patients (38)	
	Algorithms may not consider patient preferences (39)	
	Balancing individual responsibility with communal solidarity can be challenging (51)	
	Striking a balance between societal benefits and potential harms is difficult (45)	
Responsibility (*n* = 12 studies)	Ambiguity regarding the accountable party for collected data (41, 52, 58, 62, 67, 70)	Clarify the responsible party for technology validation and outline potential consequences in case of any harm (45, 52, 65, 66)
	Lack of regulatory and ethical clarity regarding accountability, moral responsibility, and legal liability (56)	Support patient autonomy and respect their decision-making (43)
	Role confusion among healthcare professionals using AI for decision-making, necessitating a balance with their own judgments (45)	Incorporate human agency and oversight (49)
	Risk of excessive reliance or complacency induced by AI tools (56)	Establish clear agreements with IT providers regarding update and security responsibilities (67)
		Informant patients that the data they generate at home will influence their physician's clinical decisions (62)
		Ensure patients are aware of the extent of access they have to the technology and the associated responsibilities (62)
Validation of eHealth (*n* = 10 studies)	Lack of clear certification systems or transparent guides for assessment (42, 53)	Ensure legal clarity and ethical soundness in technology validation (42, 49)
	Uncertainty regarding the type of clinical and socio-economic evidence required from manufacturers (53)	Establish certification of medical devices and align the required clinical evidence with European Medical Device Regulation Standards (53, 63)
	Limited availability of high-quality evidence for eHealth (62)	Continuously validate eHealth technologies through clinical assessments (58)
	Difficulty in accessing complete and generalizable evidence for efficacy and effectiveness (62)	Develop a comprehensive framework with balanced regulations and innovation-friendly criteria for health technology assessment (53)
	Challenges faced by medical ethical committees in assessing novel eHealth solutions due to their unknown impact or burden (55)	Mandate manufacturers to conduct clinical safety evaluations before market release or deployment (37)
	The significant withdrawal of patients from studies can diminish the value of data analysis (59)	Base all technology components on evidence-based principles (58)
		Promote eHealth technologies with shared benefits and measurable outcomes (66)
		Utilize real-world datasets from clinical trials for evidence generation and impact assessment (53)
Monitoring and Follow Up of Data Output (*n* = 7 studies)	The abundance of health technology choices and rapid innovation poses challenges among healthcare professionals (62, 69)	Emphasize that AI should complement rather than replace healthcare professionals, shifting their roles from processors to expert overseers (56)
	Use of technologies that upload and share data may give individuals a sense of being under surveillance (51)	Ensure healthcare professionals have prompt access to information to enhance the speed and quality of their care decisions (60)
	Technologies may result in healthcare professionals feeling obligated to be available or responsible all the time (52)	Establish a technology that issues warnings at the organizational level rather than targeting individual healthcare professionals (52)
	False alerts generated by health technology (43)	
Liability (*n* = 6 studies)	Concerns about the potential legal liability for harm to a patient's health (62)	Promote transparency regarding accountability (39, 65)
	Lack of clarity in legislation and regulations concerning liability and accountability for both producers and healthcare providers (57)	Provide guidance on responsibilities and liabilities when different components interact with each other (48)
	Manufacturers’ concerns about potential liability due to external communication infrastructure vulnerabilities that could lead to damages (57)	
	Absence of accountability for the accuracy and correctness of shared data (51)	
Implementation and Compliance with ethical policy, guidelines, and frameworks (*n* = 3 studies)	The typical industry practice of rapid prototype development with iterative cycles may not align with ethical standards (40)	Enhance the adaptability of ethical frameworks (40)
		Develop regulatory and ethical frameworks for public-private partnerships (60)
		Supply specialized guidance to specify the role of digital health in clinical practice (62)

##### 3.3.2.1 Consent

Ethical concerns arise when patients are compelled to use specific eHealth technologies due to limited alternatives (47, 56), for example, in case of exclusive reimbursement by healthcare insurers. Challenges emerge when patient data is shared with other providers or third parties without explicit patient consent (47), especially when dealing with different infrastructures and varying ethical standards across locations (35). Ensuring acceptance and consent from both patients and medical professionals is a significant hurdle (70). Granting parental access to children's medical records raises valid concerns about privacy and (45), highlighting potential privacy implications and the risk of estranged parents accessing sensitive information about themselves in the child's data. Obtaining consent can demand substantial time and effort (40), and users often overlook consent details, clicking “agree” without comprehensive understanding (51). To facilitate implementation, information disclosure can help alleviate insecurities (42, 43, 49, 56, 58, 63, 65). This entails providing operational details about the technology (49), data access and handling processes (65), associated risks and benefits (63), and access parameters (45). Furthermore, explicit consent should be obtained for data sharing (36, 45, 47, 49, 56, 63), ensuring a favorable benefit-harm ratio for patient and their caregivers (45). This enables informed decisions and prevents ambiguous consent, ensuring compliance with legal data processing confidentiality (36). Optimizing the consent process (36, 53), incorporating consent as a design element, and early digitalization (36) streamline procedures. Supportive models or platforms enhance consent management (53) and delivering information in an understandable format enhances patient empowerment (45).

##### 3.3.2.2 Transparency of data

Patients' limited awareness of the storage and sharing of their (sensitive) data poses a barrier (51, 66). “*There seems to be an issue concerning transparency with respect to insurers’ data-sharing apps, since the entire range of purposes for which users’ data is processed is not equally disclosed*” (51). The opacity of AI algorithm logic, including insufficient disclosure of methodology in healthcare AI systems (39), hampers understanding and trust (39, 45, 56, 63). Transparent communication about the methodology for assessing data quality is imperative to facilitate implementation (63, 65). Advocating for “open source” development enhances information quality (58, 65). Identifying stakeholders across various stages of technology development, including requirements, design, implementation, and operational decisions is imperative (45). This process introduces complexity due to the multiplicity of stakeholders involved across distinct stages, each driven by disparate interests. Involving all pertinent stakeholders in decision-making, potential adoption strategies, and discussions surrounding the parameters governing data utilization facilitates implementation (53, 60).

##### 3.3.2.3 Inclusiveness and diversity

Favoring users who willingly share health-related data, such as insurers providing monetary rewards (51), can pose a barrier to successful implementation (51). The ethical acceptability of offering economic incentives for data sharing is a subject of ongoing debate (51). Achieving equity in health resource allocation may raise legal considerations to ensure equal access to optimal care, regardless of patients' geographic location (69). Concerns extend to individuals with disabilities and the elderly, who may face digital literacy challenges, potentially excluding them from digital health benefits (60). Balancing individual responsibility and collective solidarity (51), along with considering societal benefits and potential drawbacks (45) is a challenge. For instance, research findings may not yield immediate advantages to the individuals whose data is used, however, they have the potential to improve healthcare for a broader population (45). Facilitating inclusive and diverse implementation involves the development of non-discriminatory technologies (43, 47, 49, 65) that are user-friendly (47, 58, 60). Moreover, ensuring the integration of individuals with specific needs or vulnerabilities within the protective framework of the social security system is pivotal (51).

##### 3.3.2.4 Responsibility

The central implementation barrier revolves around uncertainty regarding data ownership, causing confusion about accountability (41, 52, 58, 62, 67, 70, 73). The interplay between human decision-making and technology adds complexity for healthcare professionals, necessitating navigation through diverse responsibilities (45). “*Automation can muddle responsibilities as clinicians who use AI tools to support clinical decision-making may need to weigh their own judgments against those of an algorithm*” (45). Conversely, eHealth implementation can be facilitated by clarifying responsibility for eHealth technology validation and potential consequences in case of any harm (45, 52, 65, 66). “*For example, despite the [name app] is able to deliver insulin to control high blood glucose levels, it cannot provide help in case of a hypoglycemia. The responsibility of managing such events is still delegated to the patient, which must be properly trained and aware of the limits of the system*” (52). Enhancing patients' awareness of their generated data's significance in clinical decisions is pivotal (62), considering the implications of data inaccuracies, misreporting, or omissions on decision-making and patient well-being underscore this importance (62). Making patients aware of their access rights to apps or devices serves as a facilitator for implementation (62), whether access is unrestricted or controlled. Incorporating and empowering human agency and oversight is pivotal for streamlined implementation (49).

##### 3.3.2.5 Validation of eHealth

Barriers to implementing eHealth technologies encompass the scarcity of robust evidence supporting their efficacy (62), along with the absence of compelling certification systems or transparent assessment guidelines to ensure the quality of eHealth technologies (42, 53). Uncertainty surrounds the necessary clinical and socio-economic evidence for validation complicates matters (53), especially regarding organizational change (53). Acquiring comprehensive, universally applicable evidence for eHealth effectiveness remains difficult (61), and ethical committees struggle with establishing positions due to the uncertain impact and potential burdens of novel eHealth solutions (55). Facilitating implementation involves creating a framework and assessment criteria that balances regulation and innovation achieved through efficient, realistic and transparent assessment and evaluation (53). Addressing data collection, storage, access, and handling requirements (65), arranging medical device certification (63), ensuring continual clinical validation of eHealth technologies (58), and validating all technology components through evidence-based approaches (58) are essential steps. The required clinical evidence should align with European MDR specifications (53). Manufacturers play a pivotal role in expediting implementation by ensuring shared benefits and measurable outcomes (66), along with legal clarity and ethical correctness (42, 49), and conducting safety assessments prior to market entry or service initiation (37). Clinical trials provide an opportunity for leveraging real-world datasets for impact assessment without recruiting trial patients (53).

##### 3.3.2.6 Monitoring and follow up of data output

A barrier to successful implementation arises from healthcare professionals struggling to select and tailor eHealth technologies to patient's specific circumstances amid a continuous influx of novel solutions (62, 69). The challenge is heightened by the pressure to embrace the latest advancements. Technologies enabling data upload and sharing may raise concerns about surveillance, especially when health insurers scrutinize customer health behaviors (51). Critical alerts or patient communication via digital tools can make healthcare professionals feel constantly obligated to be available or responsible (52). Facilitators for implementation include emphasizing that eHealth should complement rather than replace healthcare professionals (56), enabling proper information access for faster and higher-quality decision-making (60), and directing technology warnings to a central point, such as on hospital or organizational level, instead of targeting specific healthcare professionals (52).

##### 3.3.2.7 Liability

A prominent implementation barrier emerges from unclear legislative regulations regarding liability and accountability for both producers and healthcare professionals (57). For instance, manufacturers often adopt closed-system product designs, leading to interoperability challenges among digital health devices due to unique software and arbitrary communication protocols (57). Apprehensions about accepting liability for potential harm to patient well-being can hinder the full embrace of digital health innovations in clinical practice (62). The absence of liability frameworks for the precision and fidelity of shared data (51) further complicates the situation. Patients may input behaviors or sensations into the device or app that deviate from reality, raising questions about the accuracy of self-tracking technology data (51). Conversely, a facilitator for implementation involves fostering transparency regarding responsibilities, promoting a robust sense of accountability (39, 65). Guidance on responsibilities and liabilities should be provided for different components of technologies when they interact with each other (48).

##### 3.3.2.8 Implementation and compliance with ethical policy, guidelines and frameworks

A barrier arise from the discrepancy between the industry's practice of rapid prototype development with iterative cycles and the stringent ethical requisites, especially in patient-oriented applications requiring precise technological descriptions for regulatory approval (40). Strategies to facilitate implementation include establishing well-defined parameters for public-private relationships (e.g., based on the principles of accountability, consistency, engagement, reasonableness, reflexivity, transparency, and trustworthiness) (60). These frameworks would entail explicit agreements on acceptable data usage, potential data commercialization, and ownership of intellectual property. Providing professional guidance is essential to define the role of digital health in clinical practice (62). Ethical frameworks must be adaptable (40), recognizing that existing guidelines often treat all healthcare data homogeneously despite varying degrees of sensitivity.

#### 3.3.3 Financial aspects

The included studies showed multiple financial barriers for implementation with topics related to the lack of funding or reimbursement, and business modeling. See Table 5 for an overview of all identified barriers and facilitators of financial aspects.

**Table 5:** Overview of the identified **financial** barriers and facilitators.

Topic		
	Barriers	Facilitators
Business modeling (*n* = 15 studies)	Insufficient integration of eHealth development into global business models (42)	Invest in early and effective collaboration with stakeholders (including third parties) (40, 53–55, 65, 67, 70)
	Absence of a viable business model for preventive interventions (58)	Create a financially sustainable business model (54, 58, 67)
	High financial investments are required for business models for eHealth (53)	Invest more in developing public-private partnerships (53, 60)
	Complexity arising from diverse reimbursement and funding systems across countries impeding commercialization of eHealth technologies (47)	Provide appropriate incentives to stakeholders, including users (40)
	Double payment issue: Public hospitals supplying data to private manufacturers for free, resulting in additional costs for innovation access (39)	Ensure third parties integrating health data understand the data limitations (45)
	Inadequate resources for establishing and sustaining IT infrastructure to support AI processes (70)	Consider commercialization through licensing agreements post-CE certification and clinical effectiveness evidence via RCT studies (53)
	Uncertainty in the management and maintenance of eHealth technology (67)	Develop eHealth infrastructures and human capacities for long-term reusability and improvement in centers of excellence, rather than project-specific use (70)
	Lack of clarity regarding the value proposition of eHealth for patients (58)	Ensure the technology is both technically and socially sustainable (49)
		Consider agile business models like pay-for-use or app prescriptions as alternatives to traditional licensing-based revenue (53)
		Foster innovation (60)
Reimbursement of eHealth (in practice) (*n* = 10 studies)	Challenges in assessing the cost-effectiveness, clinical benefits, and intangible impacts of health technology (41, 54, 66, 70)	Promote value-based approaches and economic modeling for health technology reimbursement to ensure long-term viability (41, 42, 53)
	Uncertainty about long-term, sustainable guarantee of reimbursement (58, 67)	Secure funding and support for early-stage technology implementation and testing to build evidence for reimbursement (42, 53)
	Lack of structured financial reimbursement mechanisms for the health technology (64)	Enhance transparency in information sharing and communication among stakeholders to support reimbursement (53, 66)
	Challenges in implementing reimbursement models spanning multiple healthcare sectors (54)	Facilitate information sharing on health technology costs between the healthcare systems and technology providers (53)
	Funding misalignment between healthcare department budgets hindering cooperation between health insurers and healthcare providers (54)	Streamline the CE marking and certification process for health technologies to expedite reimbursement (53)
	Challenges in navigating insurance complexities, cost-benefit balancing, and risk selection (66)	Create assessment frameworks that offer temporary reimbursement for CE marked technologies (53)
	Excessive costs related to improving and maintaining data validity, security, and storing (70)	Develop economic models and quality certification systems to support reimbursement (42)
		Explore the possibility of new hospital payment regulations, such as the “Optional reimbursement scheme” (54)
		Collaboratively allocate financial risk between healthcare providers and insurers (54)
		Demonstrate cost-effectiveness to attract health insurers’ interest (67)
		Increase the patient population to make eHealth projects financially viable (54)
		Specify that reimbursement for health technologies requires a prescription from a health professional (53)
		Implement shared savings and bundled payment models to incentivize cost-efficient and high-quality care (54)
		Utilize scalable cloud storage and server capacities to manage analysis needs efficiently and reduce computing costs (70)
Lack of funding for eHealth development and implementation (*n* = 5 studies)	Insufficient funding available for the integration of novel technologies into healthcare (41, 58)	*Facilitators Not Identified*
	High development costs for novel technologies (42, 69)	
	Many development ideas are often not financially viable (42)	
	Limited willingness to invest for digital health solutions (66)	
	Uncertainty regarding responsibility for covering service costs (66)	

##### 3.3.3.1 Business modeling

The lack of alignment between eHealth development and established global business models, hindering seamless integration into broader economic frameworks (42). Diverse reimbursement funding systems across countries impeding commercialization of eHealth technologies (47), and the substantial financial requirements associated with these business models pose a significant challenge favoring larger players and potentially excluding smaller stakeholders (53). Establishing financially sustainable business model serves as a facilitator for implementation (54, 58, 67). Robust public-private partnerships enhance care processes, stimulate research and innovation, and expedite technological development (53, 55, 60). Collaborating closely with commercial partners and addressing stakeholders' needs stands paramount, necessitating tailored incentives to match efforts and navigate benefit-costs challenges (40, 58). Early and inclusive involvement with stakeholders (40, 53–55, 65, 67, 70), encompassing legal specialists and data protection officers, is of importance to guide appropriate decision making (65). Licensing agreements (63), certification of health technologies as CE-devices (53), and innovative business models, such as pay-for-use or app prescriptions, introduce dynamic alternatives to conventional licensing-based revenue streams (53). Ensuring sustained success involves adaptable mechanisms for pricing and reimbursement (53). Developing eHealth infrastructures and human capacities for long-term reusability and improvement is essential, emphasizing ethical considerations and long-term viability (49, 70).

##### 3.3.3.2 Reimbursement of eHealth usage

The absence of structured financial reimbursement (64) and uncertainties surrounding guaranteed reimbursement (58, 67) are prominent barriers, compounded by challenges in measuring cost-effectiveness, costs justification, and finding a balance between costly treatments and their broader societal implications (41, 54, 66, 70). Another significant barrier revolves around the intricate challenges in navigating insurance complexities (66). “*Private* vs. *mandatory insurance, for example, risk selection on the basis of available personal data. Even though legally this is not possible, it is happening unofficially*” (66). On the contrary, value-based approaches and economic modeling for reimbursement are facilitators for implementation, by ensuring long-term viability (41, 42, 53). Economic models and quality certification systems support financial strategies for enduring eHealth viability (42). Assessment frameworks should provide temporary reimbursement for CE marked technology, enabling limited market entry for data collection supporting clinical and health economic evaluations (53). A direct connection between certification and reimbursement, along with a certification-triggered mechanism for swift price setting and reimbursement (53), facilitates implementation. Clarifying benefits and cost impacts for users, including service fees, strengthens the rationale for reimbursement decisions (66). Reimbursement of health technologies should necessitate a prescription from a health professional, reinforcing informed medical decisions (53). Transparency from insurers (66) and demonstrating cost-effectiveness (67) can attract interest and financial support for eHealth. Manufacturers should be enabled to collect real patient data with appropriate reimbursement, fostering evidence-based evaluation and collaboration (53).

##### 3.3.3.3 Lack of funding for eHealth development and implementation

Identified barriers encompass limited funding for the seamless adoption of emerging technologies (41, 58), as well as a reluctance to allocate (financial) resources (66). The financial burden of developing novel technologies compounds these challenges (42, 69). The lack of comprehensive scientific evidence on remote monitoring efficacy complicates cost justification (41), often linked to the non-viability of development concepts due to financial constraints (42). Ambiguity in financial responsibility allocation for remote monitoring services presents an additional barrier (66). These barriers collectively culminate in a national resistance to enacting substantial policy adjustments aimed at integrating standard reimbursement processes into the incorporation of remote monitoring systems (41).

#### 3.3.4 Technological aspects

The included studies showed multiple technological barriers for implementation with topics related to interoperability and integration, malfunction and errors, data reliability, the layout, and accessibility. See Table 6 for an overview of all identified barriers and facilitators of technological aspects.

**Table 6:** Overview of the identified **technological** barriers and facilitators.

Topic		
	Barriers	Facilitators
Interoperability and integration (*n* = 17 studies)	Inadequate data connectivity between IT-systems (including different versions of one system) (45, 47, 50, 53, 55, 58, 61, 66, 67, 69, 70)	Develop health technologies that seamlessly integrate with existing workflows and generate interpretable data for clinicians (40, 58, 67)
	Lack of integration between health technology and electronic medical records (50, 53, 64, 68)	Establish an eHealth infrastructure rather than standalone health technologies (60, 67, 68)
	Challenges integrating health technology with healthcare professional work practices (45, 53, 67)	Create regulatory environments that encourage integration across data sources without stifling innovation (40)
	Complexities in ensuring that health technology interoperability complies with legal system and protection legislation (67, 68)	Incorporate a connection between electronic patient files and health technologies (67)
	Non-transferable data across countries (70)	Enable seamless information exchange among healthcare providers within and between healthcare facilities (45)
	Difficulty achieving interoperability within complex healthcare organizations (60)	Align and link data from various disparate sources of origins (59)
		Utilize the potential of AI to automate data capture, distribution and communication (45)
Data reliability (*n* = 11 studies)	Discrepancies in how clinicians record or interpret data (52, 56, 59, 61, 70)	Ensure honest, accurate, and conscientious data entry (39, 45)
	Bias in the (trained) dataset (45, 47, 62, 63, 70)	Utilize quality assessment tools and adhere to quality standards (70)
	Possibility of data loss or delay (52)	Improve data reporting by using standardized reporting guidelines (70)
	Data deletion complexities in AI contexts; algorithms do not forget like humans (63)	Prioritize the reliability of data communications (52)
	AI's limited ability to differentiate causation from correlation (63)	Managing missing and unstructured data (70)
	Health technology's narrow focus might be unsuitable for defining the total health status of a patient (51)	
	Uncertainty about the appropriate incorporation of patient-initiated digital health data into clinical decision-making (62)	
Malfunction and errors (*n* = 7 studies)	External factors may impact the health technology's performance (62)	Offer support and assistance from IT-staff (36, 62)
	Risks associated with introducing new interfaces or features that could break application functionality (40)	Ensure patient awareness for potential errors, prevention measures, and response/reporting procedures (62)
	Software errors (48)	Prohibit the addition of hardware or modification of systems software (36)
	Data acquisition might adversely affect performance (39)	Routinely update software and systems (47)
		Implement a system quality control process (45)
Accessibility (*n* = 4 studies	Health technologies inundated with excessive unsolicited data can overwhelm (clinical) users (40)	Ensure health technology interfaces are accessible for users (36)
		Offer user manuals and technical support services (45, 64)
		Minimize additional activities, time and user workload associated with the use of the health technology (36)
		Schedule data extraction from health technologies outside peak office hours (36)
		Automate data extraction from patient health records (36)
		Ensure data export functions within the organization's local network (36)
		Periodically present summarized data routine management channels (40)
Layout of eHealth (*n* = 2 studies)	Lack of clarity of the language usage (43)	Supply data and information flexibly, catering to individual contexts and roles (40)
		Adopt a user-centered design approach with close stakeholder collaboration (40)

##### 3.3.4.1 Interoperability and integration

Foremost among identified barriers is the intricate challenge of inadequate data connectivity between disparate health systems, or even across disparate versions of a health system (45, 47, 50, 53, 55, 58, 61, 66, 67, 69, 70). This requires harmonizing processes and data across heterogenous healthcare services and facilities (45, 53, 66). A significant barrier is the lack of interoperability between technologies and electronic medical records (50, 53, 64, 68), exacerbated by the current phase of EHR rollout (53). Integrating eHealth into healthcare professionals' work practices poses challenges (45, 53), including updating records management to accommodate expanded technology capabilities (45). This interoperability gap not only hampers effective data re-use (53) but also raises ethical concerns, potentially affecting patient safety and hindering the full realization of platform benefits (45). Ensuring interoperability with the current legal system and proactively designing digital health systems to adhere to data protection legislations are essential, but intricate imperatives (68). Conversely, developing tools that seamlessly integrate within established workflow and yield clinically meaningful data becomes pivotal (40, 58, 67), as does fostering a robust eHealth infrastructure coupled with regulatory environments promoting data integration while fostering innovation (40, 60, 73). The exchange of information across diverse healthcare providers and empowering healthcare professionals through training on secure system utilization are pillars in fostering integration (45). Shared frameworks spanning technical, legal, and organizational dimensions underscore the potential of digital tools and data-driven technologies in public health (68). The integration of AI holds the potential to automate data processes, heralding enhanced interoperability (45), as well as a connection with electronic patient files (67).

##### 3.3.4.2 Data reliability

Implementation barriers related to data reliability encompass challenges including limited consistency in data recording practices (59, 61), errors in data interpretation (52, 59, 70), and the potential for bias arising from non-heterogenous training datasets, missing data, data loss, small sample size, underestimation, misclassification, and measurement errors (45, 47, 62, 63, 70). Inconsistencies in data recording practices among clinicians impede cohesive data interpretation, raise uncertainties about data accuracy and trustworthiness (62), and lead to reservations regarding the incorporation of patient-initiated digital health data into clinical decision-making (62), collectively obstructing the realization of efficient and reliable health insights. “*The fact remains that people could “exploit” the system to obtain monetary benefits by providing biased data or by “hiding” other unhealthy habits that are not measured (e.g., smoking)*” (51). Notably, the narrow focus of certain technologies hinders a comprehensive representation of an individual's health status (51). The limited ability of AI to differentiate causation from correlation adds an additional layer of complexity to data reliability (63). To facilitate successful implementation, a system of quality control should be instituted (70). The foundational importance of honest, accurate, and diligent data entry as a cornerstone for the quality and safety of care underscores the significance of reliable data inputs (39, 45). The implementation of system quality control mechanisms is paramount to ensure the achievement and continuous refinement of expected benefits, while safeguarding against unintended consequences (45). Ensuring the reliability of data communications emerges as essential, especially in situations where timely transmission of critical data, such as ECG signals, can profoundly impact diagnoses and clinical outcomes (52).

##### 3.3.4.3 Malfunction and errors

External factors such as internet connectivity disruptions and app malfunctions introduce barriers for implementation (62). In addition, software errors (48) and the potential impact of data acquisition on performance (39) present distinct challenges. The introduction of novel interfaces or features introduces the potential of jeopardizing application functionality (40). To facilitate eHealth implementation, a key approach involves enhancing patient awareness. By equipping patients with knowledge to anticipate, address, and report errors, especially those stemming from human actions, they are empowered to adeptly navigate potential pitfalls (62). Additionally, routinely updating software and systems (47), along with preserving the original system's structure by refraining from hardware and software alternations, safeguards data integrity and proactively mitigates hardware-related complications (36). Complementing this strategy, robust IT support encompassing accessible helpdesks and localized assistance establishes a fundamental safety net for unforeseen challenges (36, 62).

##### 3.3.4.4 Accessibility

The substantial amounts of (unsolicited) data in technologies can overwhelm the users, particularly clinicians, and reduce their engagement (40). To facilitate successful implementation, several factors should be considered. For instance, strategic scheduling of data extractions outside peak office hours minimizes disruptions and harmonizes with healthcare's diverse roles (36). Automation of data extraction from patient records and wearables streamlines the process (36). Optimizing data export within organizational networks ensures seamless information transmission (36). Blockchain could provide variable user permissions, enabling controlled data access for different stakeholders (63), such as patients authorizing specific healthcare providers. eHealth technology interfaces should be user-friendly (36), time and workload for users must be reduced to a strict minimum (36), and a user manual should be available (45, 64).

##### 3.3.4.5 Layout of eHealth

Language barriers, including unclear and non-audience-appropriate communications, pose significant hurdles to effective implementation (43). A facilitating strategy to mitigate this involve optimizing data presentation by aligning it with individual contexts and roles augments usability (40). This user-centered design approach mandates close stakeholder collaboration to maximize relevance and applicability (40).

## 4 Discussion

### 4.1 Principal findings

This scoping review aimed to provide insights into barriers and facilitators of legal, ethical, financial, and technological aspects for successful implementation of complex eHealth technologies, which impact multiple levels and multiple stakeholders.

– ***Legal barriers and facilitators*** predominantly involve preserving data confidentiality and ensuring safe and secure data management. The challenges are often compounded by vague or inadequate existing regulations. Clear governmental guidelines or frameworks are imperative for successful implementation, and they should be complemented with proactive risk and impact assessment of eHealth technologies.
– ***Ethical barriers and facilitators*** encompass the intricacies of consent, responsibility, and liability, necessitating a nuanced balance due to their interrelated nature. The validation of eHealth technologies presents considerable challenges, given the lack of clarity on assessment criteria, absence of certification systems, and insufficient clinical and socio-economic evidence. Robust ethical frameworks, aligned with legal constructs, provide guidance for navigating these challenges throughout the development and implementation of eHealth technology.
– ***Financial barriers and facilitators*** stem from insufficient funding for eHealth technology development and (post-)implementation usage, necessitating the development of business models, stakeholder involvement, and commercial partnerships for effective and sustainable implementation.
– ***Technological barriers and facilitators***, particularly interoperability and integration issues, coupled with apprehensions regarding malfunction and errors, hinder seamless implementation and adoption. Identified practical strategies to mitigate these barriers encompass ensuring data reliability, optimizing the usage of language (which entails using clear and audience-appropriate communication), layout features, enhancing technological accessibility, and aligning eHealth technology more seamlessly with existing systems and workflows.

### 4.2 Lessons learned for the implementation of eHealth

#### 4.2.1 Balancing compliance with legal and ethical regulations while fostering innovation in eHealth implementation

Existing regulations, encompassing both ethical and legal aspects, impose stringent requirements to ensure the secure and confidential storage and exchange of patient data (42, 44, 58). However, these requirements, while essential for data protection, can inadvertently stifle industrial competitiveness (44). Notably, the introduction of the Medical Device Regulations (MDR) in the EU has intensified regulations for eHealth, classifying software and eHealth technologies as medical devices if they provide advice that might influence patient behavior or treatment strategies. Depending on their intended purpose and associated risks, these devices must provide evidence of effectiveness. Moreover, stringent regulations extend beyond the medical device itself to encompass any integrated or connected components (48). The proliferation of regulations, coupled with compliance hurdles and the absence of critical infrastructure, has led companies to prioritize US market approval over the EU (44). To encourage innovation within Europe, EU health technology regulations should exhibit flexibility, enabling adaptation to evolving industry and legal frameworks. Policymakers must play an active role, considering the perspectives of both developing companies and healthcare providers (53, 70). Moreover, it is crucial to advocate for necessary adjustments in the MDR, ensuring that industry stakeholders can effectively communicate their requirements for policymakers. Periodic assessments and updates of legal and ethical requirements are imperative to ensure alignment with the evolving eHealth industry (44).

#### 4.2.2 The self-perpetuating cycle: validity and funding in the transition from eHealth research to implementation

In eHealth implementation, a recurring challenge emerges as an endless cycle intricately connecting eHealth technology validity and the availability of funding (or reimbursement). This cycle significantly impacts the transition from technology research and development to implementation for use in practice. At its core lies a paradox: eHealth must demonstrate its validity and effectiveness to gain practical acceptance and financial reimbursement. Paradoxically, potential financiers are often hesitant to support unproven technologies that lack empirical evidence of effectiveness, and ethical standards prohibit the use of eHealth without clinical validation. This paradox initiates a cycle in which the absence of initial funding hinders the acquisition of both clinical and socio-economic evidence. Conversely, the lack of evidence obstructs further funding. This cyclic challenge often leads to non-implementation or post-implementation failure of eHealth technologies due to depleted research funds. The “Valley of Death” phenomenon post-implementation—characterized by hurdles in breakthrough and scaling, such as funding deficiencies, failed technology commercialization, and insufficient governmental support for startups (74)—exacerbates the issue. Many eHealth technologies often remain at Technical Readiness Level (TRL) “3” (75), focusing on research and development, addressing mainly issues such as usability (76). However, actual implementation and scalability require advancement to TRL “7”, signifying the deployment phase, where the focus shifts to market preparation activities such as conducting clinical studies and safety assessments (76). Existing implementation frameworks, like NASSS (20), predominantly guide up to TRL “3” but fall short when contemplating the transition to TRL “7”. Our study addresses this gap by presenting a comprehensive spectrum of factors that require consideration for eHealth's transition from development to deployment. During this transition, the focus extends to sustainable implementation, scalability, legal compliance, funding and reimbursement, and ethical considerations. This underscores the significance of our review in addressing the complex challenges of the transition and enhancing comprehension of eHealth implementation dynamics.

#### 4.2.3 Navigating uncertainties in responsibility and accountability in eHealth implementation

Regulatory and ethical frameworks often lack clarity in delineating responsibilities and accountabilities of diverse stakeholders (41, 52, 58, 67, 70), leading to uncertainties during eHealth implementation and everyday usage. Healthcare professionals, for example, wrestle with concerns about their roles when eHealth pose potential harm to patients or when timely responses based on eHealth-derived information are not realized. A similar lack of clarity surrounds the extent of liability for manufacturers of eHealth in these contexts. This uncertainty is exacerbated by the significant paradigm shift in Europe, moving from institution-centric care to home-based care models (75, 77). This shift places a greater burden of responsibility on technology users, including patients and involved healthcare professionals. The shift, coupled with the reevaluation of conventional healthcare funding models and the potential bias in self-reported data, introduces new challenges in determining responsibility and accountability. Additionally, when eHealth technologies rely on AI or machine learning, providing user action suggestions, issues of accountability become paramount. Recognizing these ongoing changes underscores the imperative need to effectively consider the identified barriers and factors concerning responsibility and accountability.

#### 4.2.4 Business modeling gaps and lack of reimbursement mechanisms for financial sustainability in eHealth implementation

Reimbursement mechanisms are pivotal for ensuring financial sustainability of new eHealth solutions, yet they remain insufficiently explored in Europe (26, 78). Reimbursement pathways for eHealth vary significantly across European countries due to disparities in national laws and regulations (78). The lack of robust business modeling within the healthcare sector exacerbates these challenges. Unlike other industries, the adoption of business modeling in healthcare is relatively new (72). The diverse financing models within the healthcare domain make this challenge even more significant. Unlike traditional healthcare settings where reimbursement primarily comes from insurance providers, the eHealth domain requires a distinct approach to business modeling that must effectively serve both sides of the market (72). Various stakeholders—including both public and private entities—participate in eHealth development and implementation, each with distinct expectations and values. The diverse expectations highlight the importance of adaptable business models, as the value proposition of stakeholders is crucial for fostering eHealth innovation (72). Researchers and developers are often naïve about how a successful eHealth technology can enter and thrive in the market without support from an entrepreneur who needs to sustain it. Even in countries with robust public healthcare systems, commercial firms and entrepreneurs (third parties) play pivotal roles in driving innovation and renewal (74). However, reliance on external funding sources introduces complexity and risk due to potential conflicts with regulatory frameworks governing patient data.

### 4.3 Recommendations

This study explored the complex interplay of legal, ethical, financial, and technological considerations. While these factors often overlap, they maintain distinct identities demanding individual attention. However, they are not static entities; recognizing their dynamic interconnectedness is crucial for comprehensive evaluation, as choices in one domain can significantly impact others. Our key message is clear: effective decision-making in implementation requires active and holistic collaboration with a spectrum of stakeholders. Meticulous identification of suitable stakeholders and defining their “stake” and value propositions are paramount, involving regulatory bodies, policymakers, industry or technology experts, payers (commercial or third parties), funding institutions, ethicists, and users (such as patients and healthcare professionals). These stakeholders ensure strategies are developed with invaluable expertise and guidance for secure data management, technological advancement, ethical frameworks, funding and reimbursement capital, validation, and addressing interoperability challenges for seamless integration into clinical practice.

Building on stakeholder engagement, a pivotal strategy for sustained success entails collaboratively crafting a comprehensive business model. This strategic approach not only promotes a clear understanding of potential implementation challenges but also serves as a crucial step in mitigating the risk of encountering the “valley of death” phenomenon (74). Sustainable eHealth implementation relies on robust and innovative financial support. However, the highly heterogeneous eHealth market introduces significant costs and risks when targeting diverse niches, often causing providers to lack the necessary resources for the development of large-scale infrastructures. This limitation narrows business model options to stand-alone, single-function equipment, complicating installation, maintenance, and use (26). Additionally, the market's fragmentation hampers innovation, limits interoperability, and hinders the full realization of network externalities' benefits (26). To secure the sustainable future of eHealth, we should shift from developing isolated standalone tools to establishing a comprehensive infrastructure (14, 60, 67, 68, 70). Developing eHealth requires a digital health environment, encompassing multidisciplinary collaborations, the establishment of legal and ethical frameworks, and seamless interoperability for data exchange between systems and individuals. Furthermore, eHealth technologies should be designed with extensibility in mind to address the challenges posed by the relatively small market size (26). This multifaceted approach aligns with the diversity of the eHealth market and is essential to consider in business models for fostering innovation, enhancing interoperability, and ensuring comprehensive services and long-term maintainability (26). Besides stakeholder engagement, involving a diverse user group in the development process is imperative. This inclusive approach ensures eHealth technologies meet diverse user needs, improving usability, and contributing to long-term maintainability.

**Figure 3:**
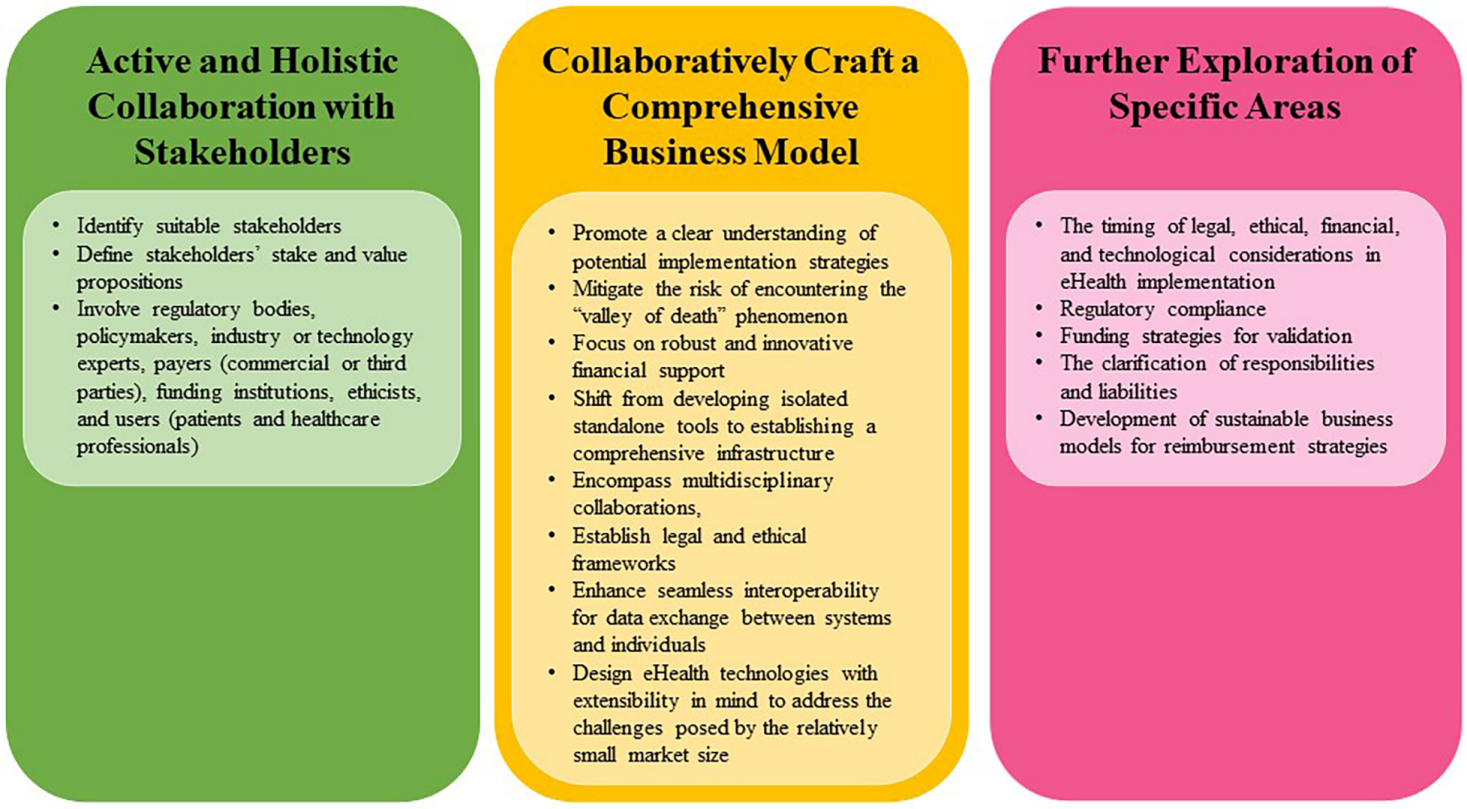
Overview of all recommendations from this study.

As we have gained a comprehensive understanding of the critical factors surrounding legal, ethical, financial, and technological considerations in eHealth implementation, future research should explore the timing of these considerations. Additionally, we identified specific areas from our lessons learned, such as regulatory compliance, funding strategies for validation, the clarification of responsibilities and liabilities, and the development of sustainable business models for reimbursement strategies, which require further exploration. An overview of all our recommendations is shown in Figure 3.

To delve deeper into these complex issues, future research will extend beyond peer-reviewed work, including insights from non-peer-reviewed and gray literature—including industry reports and policy documents—to enhance our comprehension and strategy development for the legal, ethical, financial, and technological considerations. Furthermore, our upcoming research endeavors will include stakeholder workshops, featuring the eHealth Junior Consortium as a prominent case study, working to establish a digital health environment for chronically ill children (29). Our ongoing research initiatives will culminate in creation of a comprehensive roadmap for implementation, that not only covers the considerations identified from the current and planned future studies but also demonstrates the intricate cross-factor interplay, the optimal timing for decision-making, and the pertinent stakeholders associated with each facet. Alongside with this, we will develop a robust business model, using the Business Canvas Model (79) as our framework. By leveraging the insights gained from this study and acknowledging the diversity of implementation stages, we aim to provide a valuable tool for navigating the complex field of eHealth implementation.

### 4.4 Limitations

Our study reveals significant lacunae in our understanding of barriers and facilitators, underscoring a lack of documented knowledge rather than an absence of these factors in the legal, ethical, financial, and technological aspects of implementation. Particularly underexplored are facilitators, amplified by the novelty of this subject. The evolving nature of eHealth technologies further complicates the quest for effective approaches to implementation. For instance, fast developments result in limited comprehensive studies on medical devices, as well as the regulations on AI that will come soon. Within our study, we encompass the European context, yet our insights offer a globally applicable foundation. Regional legal disparities may influence implementation decisions on different continents, underscoring the importance of scrutinizing cross-regional legal and regulatory variations. While we categorize barriers and facilitators into legal, ethical, financial, and technological aspects, factors often span multiple dimensions, such as responsibility, privacy, and validation. However, our study illuminates influences, yet complex interactions among these factors demand further exploration. In addition, the use of predetermined data-driven stopping criteria in our second round may introduce a limitation in potentially excluding relevant studies. To address this concern, we employed a comprehensive manual assessment in the initial round as input for the AI learning process, and we set a higher stopping criterion of 150 consecutive irrelevant studies. Our method's strength lies in heterogeneity, providing unique perspectives on types of eHealth and their diverse phases of implementation. This diversity might challenge unified conclusions, although our findings allow us to infer the spectrum of factors requiring considerations.

### 4.5 Conclusions

This study emphasizes the growing significance of digital health environments within the domain of eHealth while revealing a critical knowledge gap in comprehending and addressing the legal, ethical, financial, and technological challenges inherent in implementing these complex eHealth technologies. Our findings provide vital insights into the multifaceted considerations essential for successful eHealth implementation. Clear guidelines and government support are imperative in the legal domain for secure data management. Robust multidisciplinary frameworks are required to address ethical considerations, encompassing issues regarding consent, responsibility, and liability. Innovative funding strategies (including public-private partnerships) and adaptable business models are crucial to tackle financial challenges. Practical solutions to enhance interoperability and facilitate data exchange are needed for addressing technological considerations. To achieve successful implementation, we can conclusively state that a multidisciplinary based, holistic and collaborative engagement with a spectrum of stakeholders is paramount, serving as the cornerstone of effective decision-making across all pertinent considerations This research underscores the pivotal transition from standalone eHealth tools to the indispensable integration of legal, ethical, financial, and technological aspects, collectively forming the comprehensive framework of digital health environments. The identification of suitable stakeholders, coupled with a clear recognition of their stakes and value propositions, ensures that implementation strategies are enriched by invaluable expertise and guidance across all aspects of eHealth implementation. Future research should explore the timing of these considerations and seek practical solutions for regulatory compliance, funding strategies (including validation), responsibility and liability, and business modeling for reimbursement strategies. Building upon the insights from this study, augmented by a comprehensive exploration of non-peer-reviewed literature, and in collaboration with forthcoming stakeholder workshops, we endeavor to craft a valuable comprehensive roadmap for navigating the intricate field of eHealth implementation. This roadmap will also be enriched with considerations of organizational and human factors of implementation.

## Data Availability

The original contributions presented in the study are included in the article/Supplementary Material, further inquiries can be directed to the corresponding author.
